# Infective endocarditis caused by HACEK group bacteria—a registry-based comparative study

**DOI:** 10.1007/s10096-021-04240-3

**Published:** 2021-04-14

**Authors:** Anna Bläckberg, Christian Morenius, Lars Olaison, Andreas Berge, Magnus Rasmussen

**Affiliations:** 1 Division of Infection Medicine, Department of Clinical Sciences, Lund, Lund University, BMC B14, SE-221 84 Lund, Sweden; 2 Department of Infectious Diseases, Skåne University Hospital, Lund, Sweden; 3 Department of Infectious Diseases, Institute of Biomedicine, University of Gothenburg, Gothenburg, Sweden; 4 Swedish Society of Infectious Diseases, Uppsala, Sweden; 5 Unit of Infectious Diseases, Department of Medicine, Solna, Karolinska Institutet, Stockholm, Sweden; 6 Department of Infectious Diseases, Karolinska University Hospital, Stockholm, Sweden

**Keywords:** HACEK, Infective endocarditis, Antibiotic therapy, Pathogen

## Abstract

Infective endocarditis (IE) caused by bacteria within *Haemophilus* (excluding *Haemophilus influenzae*), *Aggregatibacter*, *Cardiobacterium*, *Eikenella* and *Kingella* (HACEK) is rare. This study aimed to describe clinical features of IE caused by HACEK genera in comparison with IE due to other pathogens. Cases of IE due to HACEK were identified through the Swedish Registry of Infective Endocarditis (SRIE). Clinical characteristics of IE cases caused by HACEK were compared with cases of IE due to other pathogens reported to the same registry. Ninety-six patients with IE caused by HACEK were identified, and this corresponds to 1.8% of all IE cases. Eighty-three cases were definite endocarditis, and the mortality rate was 2%. The median age was 63 years, which was lower compared to patients with IE caused by other pathogens (66, 70 and 73 years respectively, *p* ≤ 0.01). Patients with IE caused by *Haemophilus* were younger compared to patients with IE due to *Aggregatibacter* (47 vs 67 years, *p* ≤ 0.001). Patients with IE due to HACEK exhibited longer duration from onset of symptoms to hospitalization and had more prosthetic valve endocarditis compared to patients with IE due to *Staphylococcus aureus* (10 vs 2 days, *p* ≤ 0.001, and 35 vs 14%, *p* ≤ 0.001). This is, to date, the largest study on IE due to HACEK. *Aggregatibacter* was the most common cause of IE within the group. The condition has a subacute onset and often strikes in patients with prosthetic valves, and the mortality rate is relatively low.

## Background

The group referred to as HACEK consists of Gram-negative bacteria belonging to the genera of *Haemophilus* (excluding *Haemophilus influenzae*), *Aggregatibacter*, *Cardiobacterium*, *Eikenella* and *Kingella*. These bacteria are gram-negative commensals of mainly the oral cavity that on rare occasions cause infective endocarditis (IE) [1, 2]. IE is an infection to the heart valves and is an important differential diagnosis in patients presenting with unspecific systemic symptoms and signs of inflammation. IE mainly affects valves with previous pathology and can lead to heart failure and septic embolization. Despite HACEK group bacteria being well-known aetiology of IE and even mentioned in the diagnostic Duke criteria [3], knowledge of HACEK-related IE emanates mainly from smaller case series. The HACEK group of bacteria constitutes only 1–3% of all IE cases [2], but growth of HACEK bacteria in blood cultures implies a 40% probability of an IE diagnosis [4]. The most extensive study of IE due to HACEK is a multinational collaboration of centres (ICE) reporting cases of IE to a registry [5]. In that study, 77 cases of IE due to HACEK were reported, constituting 1.3% of all IE cases. Cases of IE caused by HACEK were compared to those caused by all other pathogens, and it was demonstrated that patients with IE due to HACEK were younger, had a higher risk of embolic stroke and had a lower risk of heart failure compared to patients with non-HACEK-related IE. The most commonly encountered bacterial species were *Haemophilus* (40%), *Aggregatibacter* (34%), *Cardiobacterium* (14%), *Eikenella* (5%) and *Kingella* (5%). Forty percent of patients had a surgical intervention, and mortality was 11% which was significantly lower than in the control group [5]. In an older study, 45 cases of IE due to HACEK were reported in patients with a mean age of 48 years of whom 71% were male and 60% had a predisposing heart disease. A comparison group was however lacking in that study [6]. The causative organisms belonged mainly to the *Haemophilus*, *Aggregatibacter* and *Cardiobacterium* species and only two patients died from the infection [6]. In a relatively recent publication, 16 cases of IE due to HACEK, reported to a Spanish registry, were compared to cases of streptococcal IE reported to the same registry. The groups had many similarities, but the prognosis was significantly more favourable for cases of IE caused by HACEK [7].

Here we utilize a national registry of IE to describe cases of IE due to HACEK and compare these to cases of IE caused by other major bacterial pathogens.

## Methods

### Identification of bacterial isolates

The Swedish Registry of Infective Endocarditis (SRIE) is organized by the Swedish Society of Infectious Disease and holds electronical records of patients treated for IE in Sweden since 1995. Since 2008, an internet-based reporting system was instituted. All thirty departments of infectious diseases have participated since its inception. From 2018, the design of the SRIE was altered. The departments have regional responsibility for the treatment of IE, report cases treated as IE to the SRIE on a voluntary basis. Reported cases of IE caused by HACEK, *Staphylococcus aureus*, alpha-haemolytic streptococci, (including *Abiotrophia*, and *Granulicatella*) and enterococci were extracted and compared. No imputations were made when data was missing. Cases of IE reported to be caused by *Haemophilus paraprophilus* were reclassified as *Aggregatibacter aphrophilus* since this species recently changed name [8].

### Statistics

For pairwise comparisons Chi^2^-test or Fisher’s exact test was applied for categorical variables. Continuous variables were compared utilizing the Mann-Whitney *U* test. Significance was defined as a *p*-value less than 0.05. GraphPad Prism, version 8 (GraphPad Software) was utilized for statistical calculations.

## Results

### The HACEK group

Between 2008 and 2017, 5231 cases of definite or possible IE had been reported to the SRIE according to the modified Duke criteria [3]. Some cases, occurring in 2018–2019, were included as they were reported in the older system designed before 2018. Ninety-six cases caused by HACEK were identified which corresponds to 1.8% of total cases. Based on the Swedish population (from 9.3/10.2 million during the period), the incidence of IE due to HACEK was approximately 1 IE/10^6^ annually. *Aggregatibacter* was the most common causative genus, and the distribution of genera and species is given in Table 1. Eighty-three cases were definite endocarditis. One case of IE due to *H. influenzae* was included as the species was reported as the cause of IE due to HACEK in the registry. Table 2 summarizes clinical features of IE caused by the different HACEK group genera. Statistical comparisons were only made between cases of IE caused by *Haemophilus* and *Aggregatibacter*, since the other groups were small. The only statistically significant difference between those two groups was younger age for patients with IE caused by *Haemophilus* versus *Aggregatibacter* aetiology (47 vs 67 years, *p* ≤ 0.001). Predisposing factors for IE were recorded in 17 (71%) patients with IE due to *Haemophilus* and in 36 (73%) patients with IE caused by *Aggregatibacter*. Two patients experienced a relapse of IE both of which had definite endocarditis due to *Haemophilus* and *Aggregatibacter* respectively. The patients experienced native valve endocarditis (NVE) and prosthetic valve endocarditis (PVE).

**Table 1:** HACEK pathogens causing infective endocarditis

HACEK pathogens	*n* (%)
*Haemophilus* spp.	24 (25)
*Haemophilus parainfluenzae*	23 (96)
*Haemophilus influenzae*	1 (4)
*Aggregatibacter* spp.	49 (51)
*Aggregatibacter actinomycetemcomitans*	28 (57)
*Aggregatibacter aphrophilus*^a^	16 (33)
*Aggregatibacter* sp.	5 (10)
*Cardiobacterium* spp.	13 (14)
*Cardiobacterium hominis*	8 (62)
*Cardiobacterium valvarum*	2 (15)
*Cardiobacterium* sp.	3 (23)
*Eikenella corrodens*	3 (3)
*Kingella kingae*	7 (7)
Total	96

^a^Three species were reclassified from *Haemophilus paraprophilus* to *Aggregatibacter aphrophilus* according to current taxonomy

**Table 2:** Clinical characteristics of cases of IE within the HACEK genera

	*Haemophilus* *n* = 24	*Aggregatibacter n* = 49	*Cardiobacterium n* = 13	*Eikenella n *= 3	*Kingella* *n* = 7
Age, years, median	47 (30–60)	67 (55–73)^***^	64 (53–75)	69 (60–69)	59 (32–65)
Gender (% m)	14 (58)	33 (67)	13 (100)	3 (100)	5 (71)
Number of definite IE	23 (96)	44 (90)	8 (62)	2 (67)	6 (86)
Underlying disease					
Diabetes	1 (4.2)	3 (6.1)	0 (0)	0 (0)	1 (14)
Cancer	0 (0)	2 (4.1)	0 (0)	0 (0)	0 (0)
Underlying heart disease					
Native valve disease	9 (38)	11 (22)	2 (15)	2 (67)	2 (29)
Prosthetic heart valve	7 (29)	16 (33)	7 (54)	2 (67)	2 (29)
Previous IE	2 (8.3)	2 (4.1)	3 (23)	1 (33)	1 (14)
Pacemaker/ICD	4 (17)	13 (27)	2 (15)	0 (0)	1 (14)
Predisposing factors for IE	17 (71)	36 (73)	10 (77)	2 (67)	4 (57)
Type of infection					
NVE, left isolated	12 (50)	19 (39)	6 (46)	1 (33)	5 (71)
NVE, right isolated	1 (4.2)	2 (4.1)	0 (0)	0 (0)	0 (0)
NVE, double sided	0 (0)	1 (2.1)	0 (0)	0 (0)	0 (0)
PVE	8 (33)	15 (30)	7 (54)	2 (67)	2 (29)
CIED	3 (13)	12 (24)	0 (0)	0 (0)	1 (14)
Location of acquisition					
Community	23 (96)	46 (94)	13 (100)	2 (67)	7 (100)
Nosocomial	0 (0)	3 (6.1)	0 (0)	1 (33)	0 (0)
Health care associated	1 (4.2)	0 (0)	0 (0)	0 (0)	0 (0)
Course of disease					
Onset to hospitalization, d	7 (5–38)	19 (5–15)	16 (2–68)	2 (0–4)	7 (4–13)
Length of stay, d	31 (28–36)	33 (22–42)	33 (22–52)	42 (18–86)	25 (14–37)
Treatment length, d	34 (28–42)	32 (29–43)	30 (28–42)	42 (0–70)	28 (25–42)
Embolization	6 (22)	10 (22)	3 (23)	1 (33)	0 (0)
Surgery	11 (40)	14 (30)	8 (62)	0 (0)	3 (43)
Mortality	0 (0)	1 (2.1)	1 (7.7)	0 (0)	0 (0)

Interquartile range (IQR) is presented in brackets when continuous variables are presented. Categorical data are presented as *n* (%), and continuous data are presented as median. Statistical analyses were done by comparing *Haemophilus* and *Aggregatibacter*. For categorical variables Chi^2^ or Fisher’s exact test was used, and for continuous variables the Mann-Whitney *U* test was used

*IE* infective endocarditis, *SRIE* Swedish Registry of Infective Endocarditis, *IQR* interquartile range, *ICD* implantable cardioverter defibrillator, *NVE* native valve endocarditis, *PVE* prosthetic valve endocarditis, *CIED* cardiac implantable electronic device infection, *Mortality* death during hospital treatment.

^***^p ≤ 0.001

### Antibiotic treatment

Table 3 summarizes the antibiotic treatment for patients with IE due to HACEK. Forty-seven patients were diagnosed with NVE and thirty-four with PVE. Fifteen patients had isolated cardiac implantable electronic device (CIED) infection.

**Table 3:** Antibiotic treatment of IE due to HACEK (*n* = 95)

Antibiotic	NVE *n*	PVE *n*	CIED *n*	Total *n*
Single therapy	41	29	13	83
Beta-lactam	36	16	12	64
Beta-lactam/quinolones^a^	5	10	1	16
Beta-lactam/other antibiotics^b^	0	2	0	2
Quinolones	0	1	0	1
Combination therapy	5	5	2	12
Beta-lactam and aminoglycosides	3	3	2	8
Beta-lactam and other antibiotics^c^	2	1	0	3
Several antibiotics	0	1	0	1

Data regarding antibiotic treatment was missing in one patient

*NVE* native valve endocarditis, *PVE* prosthetic valve endocarditis, *CIED* cardiac implantable electronic device infection

^a^Defines succeeding antibiotics

^b^Vancomycin or/and aminoglycoside

^c^Vancomycin or quinolones

A beta-lactam antibiotic (ampicillin or third generation of cephalosporin) was given as single therapy for a majority of patients with NVE (*n* = 36) with a median of 30 days, (interquartile range (IQR) 28–33). Five patients were treated initially with a beta-lactam, succeeded by ciprofloxacin. Combination therapy was given in five patients of whom three received aminoglycosides for a median of 45 days, (IQR 34–45).

Sixteen patients with PVE were treated with single therapy beta-lactam antibiotic for a median of 42 days, (IQR 29–45) which was statistically significantly longer compared to patients with NVE, *p* = 0.008. Combination therapy was instituted in five patients of whom three were treated with aminoglycosides for a median of 31 days (IQR 28–48).

As for isolated CIED infection, twelve patients received beta-lactam as single therapy for a median of 30 days, (IQR 25–34). Two patients were treated with combination therapy with aminoglycosides for a median of 34 days, (IQR 25–42).

### Outcome and clinical course

Surgery was performed in eighteen cases of NVE, with valvular exchange in 13 cases and valvular plastic surgery in five cases. Death during therapy occurred in one patient on treatment day 41. The patient was a 78-year-old woman with possible endocarditis due to *Aggregatibacter aphrophilus*. The patient received beta-lactam succeeded by ciprofloxacin.

Eleven patients with PVE underwent surgery, nine patients had valvular exchange and two patients had pacemaker extraction. Death occurred in one patient on day 59 due to severe aortic insufficiency, 14 days after ended antibiotic treatment. The patient was a 56 years old man with definite endocarditis due to *Cardiobacterium hominis*.

Pacemaker extraction was performed in eight out of ten patients with isolated CIED infection.

### Comparison between IE due to HACEK and IE caused by other pathogens

As comparators to the cases of IE due to HACEK, 1935 cases of IE caused by *S. aureus* (37% of all reported cases to SRIE), 1484 cases of alpha-haemolytic streptococcal IE (29%) and 538 cases of enterococcal IE (10%) were identified. Table 4 summarizes the clinical features of IE caused by HACEK and other more common pathogens. Patients with IE due to HACEK tended to be younger (*p* ≤ 0.01), have less comorbidities, (*p* ≤ 0.05) and have community acquisition of the infection to a larger extent, (*p* ≤ 0.001). A significantly larger proportion of patients with IE due to HACEK had more predisposing factors for IE compared to IE caused by *S. aureus* and alpha haemolytic streptococci, *p* ≤ 0.01 and *p* ≤ 0.05. Compared to patients with IE due to *S. aureus*, patients with IE caused by HACEK had statistically significant longer duration from onset of symptoms to hospitalization (10 vs 2 days, *p* ≤ 0.001) and were more often diagnosed with PVE (35 vs 14%, *p* ≤ 0.001). Patients with IE due to HACEK were more often subjected to surgery (39%), and the mortality was only 2% which was significantly lower than for two of the comparator groups (*p* ≤ 0.01 and *p* ≤ 0.001).

**Table 4:** Clinical features of cases of IE due to HACEK compared to other pathogens

	HACEK *n *= 96	*Staphylococcus aureus n* =1935	Alpha-haemolytic streptococci* n* = 1484	Enterococci* n* = 538
Age, years in median (IQR)	63 (47–72)	66 (46–79)^**^	70 (58–80)^***^	73 (61–72)^***^
Gender (% m)	71	62	72	76
Number of definite IE (%)	86	94^***^	90	92
Underlying disease (%)				
Diabetes	5.2	18^***^	13^*^	18^***^
Cancer	2.1	9.2^*^	10^**^	16^***^
IVDU	0	24^***^	3.6	13^***^
Underlying heart disease (%)				
Native valve disease	26	12^***^	30	19
Prosthetic heart valve	35	14^***^	26^*^	38
Previous IE (%)	9.4	8.8	9.3	16
Pacemaker/ICD (%)	20	17	7.9^***^	18
Predisposing factors for IE, (%)	72	59^**^	60^*^	70
Type of infection (%)				
NVE, left isolated	45	53	69^***^	49
NVE, right isolated	3.1	21^***^	2.4	4.8
NVE, double sided	1.0	2.1	0.5	0.2
PVE	35	14^***^	26^*^	38
CIED	16	12	3.0^***^	8.9^*^
Location of acquisition (%)				
Community	95	82^***^	92	80^***^
Nosocomial	4.2	12^*^	4.6	13^*^
Health care associated	1.0	3.7	1.3	4.6
Course of disease				
Onset to hospitalization (d)	10 (4–27)	2 (1–5)^***^	14 (3–33)	7 (2–21)^**^
Length of stay (d)	31 (23–41)	33 (27–44)	29 (18–37)^*^	39 (29–46)^***^
Treatment length (d)	32 (28–42)	30 (28–40)^*^	28 (26–34)^***^	37 (28–42)
Embolization (%)	21	44^***^	22	23
Surgery (%)	39	24^**^	22^***^	26^*^
Mortality (%)	2.1	15^***^	5.7	11^**^

Interquartile range (IQR) is presented in brackets when continuous variables are presented. Categorical variables are presented as count (percentage) and continuous data as median. Statistical analyses were done by comparing HACEK to the other pathogens respectively. Statistical tests used for categorical variables were Chi^2^-test and when the outcome was less than five Fisher’s exact test was used. For continuous variables, the Mann-Whitney *U* test was used

*IE* infective endocarditis, *SRIE* Swedish Registry of Infective Endocarditis, *IQR* interquartile range, *IVDU* intravenous drug use, *ICD* implantable cardioverter defibrillator, *NVE* native valve endocarditis, *PVE* prosthetic valve endocarditis, *CIED* cardiac implantable electronic device infection, *Mortality* death during hospital treatment

*p*-values are presented in the following way: *p ≤ 0.05; **p ≤0.01; ***p ≤0.001

## Discussion

This study is the largest one to date describing IE due to HACEK, and most of the features presented here are in line with previous findings by others. Importantly, this study confirms that IE caused by HACEK has a more favourable prognosis than IE caused by most other pathogens [5, 7]. The mortality, however, was lower in our cohort as compared to the international cohort study [5], possibly reflecting that the present study is based on data from an entire country and not only on patients treated in tertiary centres. The previous international study compared IE due to HACEK with IE caused by any other pathogen whereas we compared IE due to HACEK to IE caused by other individual pathogens. We believe that our approach is more accurate and found interesting differences mainly between IE due to HACEK and IE caused by *S. aureus*. IE caused by HACEK shared many similarities to IE caused by alpha-haemolytic streptococci but also several differences, such as increased risk for PVE or CIED infection with HACEK, were noted. Comparisons of clinical characteristics were made between the HACEK group and the other major bacteria. The proportion of IE cases caused by HACEK was higher in our cohort as compared to the international study, perhaps also reflecting the lower selection bias in our nationwide cohort.

As in previous studies, patients with IE due to HACEK were younger compared to those with IE caused by other pathogens. The comorbidities were relatively few, and the onset of the infection was subacute. Interestingly, since our cohort is relatively large, we could compare patients with IE caused by different HACEK genera and found that those infected by *Haemophilus* were significantly younger compared to those infected by the other genera. The reason for this interesting observation is not evident from the results of the present study. Other than age differences, the material was too small to draw conclusions about possible differences between IE caused by different HACEK genera. Similar to other studies, IE caused by *Haemophilus*, *Aggregatibacter*, and *Cardiobacterium* were common whereas *Eikenella* and *Kingella* were rare causes. Quite recently, the taxonomy of the HACEK group was updated and the genus *Aggregatibacter* now also encompasses species formerly known as *Haemophilus aphrophilus* and *Haemophilus paraprophilus* [8]. This might explain the fact that older studies indicated *Haemophilus* as a common pathogen within the HACEK group [9] whereas contemporary studies, including the present one, suggest *Aggregatibacter* to be the most common HACEK pathogen in IE [10]. In our study, the majority of cases were treated with a single antibiotic, typically a cephalosporin or ampicillin, while combination therapy was given to only 12% of patients. The mortality and relapse rates were relatively low, and our results indicate that single antibiotic therapy is sufficient in most cases. In a recent study on HACEK bacteraemia, also from Sweden, one relapse was identified during a 1-year follow-up [11]. However, we cannot exclude that some patients in our study might have suffered late relapses since follow-up was recorded in 73 cases, with a median of 40 days.

An obvious limitation of this study is a potential selection bias in what patients who are reported to the SRIE. Patients who did not receive treatment at an infectious diseases department might have been missed. Moreover, only the data imputed into the database was available for analysis and no patient medical records were studied. The registry was revised in 2018, and new imputed cases of IE after 2018 can have been included as it has been imputed in another data format. Data on comorbidities were relatively scarce, and information on exact mode of species determination of bacteria was not available. Strengths of the study include the nationwide inclusion which decreases the risk for selection bias which comes from studies in tertiary centres. Another strength is the size of the study which allows for comparisons not only between HACEK and other pathogens but also within HACEK genera. We identify the need for future population-based studies with low risk of selection bias to be able to draw definite conclusions on the nature of IE due to HACEK.

## Conclusion

This is, to date, the largest study on HACEK IE. *Aggregatibacter* and *Haemophilus* were the most common causative genus where IE due to *Haemophilus* occurs in the young age group. IE caused by HACEK often strikes in patients with a valve prosthesis or pacemaker and has a subacute onset of presentation. A single antibiotic regimen is usually effective, and the mortality rate is low.
